# Comment on “Disinfection Byproducts in Drinking Water and Evaluation of Potential Health Risks of Long-Term Exposure in Nigeria”

**DOI:** 10.1155/2018/1901429

**Published:** 2018-02-20

**Authors:** James Grellier

**Affiliations:** ^1^European Centre for Environment and Human Health, University of Exeter Medical School, Truro, Cornwall, UK; ^2^Department of Epidemiology and Biostatistics, School of Public Health, Imperial College, London, UK

The article by Benson et al. [1] gives me considerable cause for concern. Overall, I would argue that the authors have not presented an evaluation of the potential health risks of long-term exposure to disinfection by-products (DBPs) in this article as they claim, as the data and methods used are not fit for purpose and the conclusions drawn are inappropriate. I found it particularly strange that the authors cite an article my colleagues and I wrote [2], which criticises the very methods that they employed in their work, apparently either without reading it or choosing to completely ignore its contents.

The authors have failed to correctly interpret the evidence linking DBPs to health; they have not addressed the complex nature of DBP occurrence and its variability in time and through the distribution network—thereby incorrectly characterising exposure—they have used rescinded cancer potency data that is incongruent with the current understanding of how chloroform may act in carcinogenesis and, perhaps most importantly of all, they have not balanced the potential risks associated with exposure to DBPs against the very real health benefits resulting from drinking water disinfection. I elaborate on a number of these points in the following commentary.

There are serious shortcomings in the exposure estimation conducted. It is true that the concentrations of DBPs measured at four Nigerian drinking water treatment plants (DWTPs) presented in the article appear to be very high relative to what is measured at the consumer tap in, for example, European countries. However, it is important to emphasise that samples in this study were taken* exclusively* at the DWTPs and not at the consumer tap. In point 5.3.1 of the Nigerian Standard for Drinking Water, it is stated that sampling should be done for centralized drinking water systems in the distribution system [3], that is, not at the DWTP; hence the comparison of measured THMs and the limits is inappropriate. The concentrations of DBPs exhibit considerable variability both temporally and geographically according to the physicochemical properties of source water, the nature of treatment and distribution systems, and climate [4, 5]. DBP concentrations are known to vary considerably across drinking water distribution networks [4]. When risks based on short term or point estimates of exposure are applied to a population in calculating lifetime cancer risks, those exposure estimates are considered valid averages for 70 years. It has been shown, however, that there is a considerable degree of variability in THM concentrations both within and between 24-hour samples [6, 7], on a seasonal basis [8], and among year-on-year averages [9]. Given the potentially high variability in concentrations, the number of samples on which the risk calculations are based is incredibly small, just three samples of treated water at each plant per month, for five months of a single year. The authors report a remarkable drop in TTHM during the study. Notwithstanding this huge variation, they go on to calculate the total lifetime cancer risk based (check this) on the very high levels found in the first part of the experiment. Why have the authors used what appears to be an absolute “worst case” scenario as the standard exposure throughout the entire lifetime of a population, particularly since they are evidently aware of the potential for very high variability over time from their own measurement data.

Confusing information is presented in the article about the species of DBP present in the water samples. The authors claim that “…average concentrations of trihalomethanes in primary and secondary water samples from the WTPs generally followed the sequence TCM > BDCM > TBM = DBCM, which was consistent with similar documented reports.” It is unclear to me what is meant by the equals sign in this ordering, but assuming this is a typographic error, they only report above detection limit data on three of the four species (TCM (trichloromethane), BDCM (bromodichloromethane),* and* DBCM (dibromochloromethane)) at one site (LW). Additionally, the order of concentrations is reported as TCM > BDCM > DBCM, which again does not correspond to the sequence they report. In any case, a set of three measurements from a single site is absolutely inadequate to make any general statement about distributions of different DBPs at all of the DWTPs that they sampled.

The authors have not sufficiently reviewed—or perhaps misunderstood—available evidence relating exposure to health effect in either human or animal studies. The authors assert that epidemiological studies have reported on health effects only among those consuming water with DBPs in excess of the maximum contaminant level (MCL). This is not the case and indicates to me that the authors have not understood the epidemiological information presented to them on the health risks associated with DBP exposure, the levels of DBPs to which those study populations were exposed, or the lack of evidence for causality in these studies. The epidemiological studies typically have found associations between various health effects and average residential DBP concentrations. Various systematic reviews, meta-analyses, and pooled analyses of cancer studies have been conducted [4, 10–13], and these show no consistent evidence of associations between DBP exposure and the majority of cancers. In terms of cancers, consistent positive associations have only been found between average residential THM concentrations and bladder cancer in men [10, 14, 15]; the concentrations of THMs measured in the majority of available studies are below MCLs. Human studies in which bladder cancer has been linked to residential THM concentrations have not been able to demonstrate that a particular chemical in the DBP mixture causes these effects: measurements of THMs are used in these studies because of the availability of monitoring data and are used as a proxy for an* unknown* putative agent.

Perhaps the chief failing of the study is the use of an oral slope factor (SF) for TCM. No reference has been provided by the authors to explain where they obtained these data. One may assume that the value given for the TCM SF was extracted from a secondary (now outdated) source, rather than from a review of the primary source (the US EPA); this has been done extensively in other literature that I have critically reviewed previously [2]. Had they used the primary source (e.g., the USEPA IRIS database), they would have found that the SF for TCM was rescinded in the early 2000s. TCM has not been considered by the US EPA as a genotoxic carcinogen since as early as 2001 [16]. Even if the SF for TCM were valid, there are several reasons why predictions of cancer risks in populations using such methods are liable to be biased. SFs are derived by modelling animal carcinogenicity data for each applicable exposure pathway for the most sensitive cancer endpoint. For a suspected genotoxic carcinogen, it is assumed that exposure at any level increases the probability of cancer. Estimates of lifetime cancer risk based on SFs are derived from the upper 95% bound of the linearized multistage (LMS) model. Application of LMS in this way generates estimates of nonzero cancer potency even when that parameter is zero [17]: as such, actual risk may be anywhere below the quoted upper bound PF and zero [18]. The USEPA has specifically employed this method in the regulatory context to set guidelines at which the excess risk of cancer is essentially too small to estimate, so that public health is protected. Benson et al.* do* state that a cancer risk calculated using SFs should be understood as an upper bound estimate. Regrettably, however, they do not present the more important point that the modelling methods used to calculate SFs results in any risk estimated using SFs (even where they are applied for appropriate chemicals) potentially being as low as zero.

The fact that an SF for TCM does not exist on the US EPA's website for use with the approach described in the paper by Benson et al. completely undermines their findings, in particular since they found only TCM in water supplies and no brominated species (with the exception of five data points). In any case, they present estimates of cancer risk for TCM (Table 5 in the article). As explained above, these cancer risk estimates have been calculated only on the basis of TCM and using an SF that should not have been applied; as such they are invalid. The mode of action through which TCM is hypothesized to operate as a carcinogen is by a nongenotoxic cytotoxic mode of action [19] wherein hepatotoxicity is a prerequisite for carcinogenesis to occur [20, 21]. The USEPA IRIS assessment of the cancer risk of TCM formally noted as early as 2001 that the PF had been rescinded because the reference dose (RfD)—based on assessment of liver toxicity—was protective for cancer. Using linear assumptions about dose-response of this chemical grossly overestimates cancer risk in this study. It is worthy of note that even application of the RfD will result in a conservative estimate of risk and according to the US EPA should be considered to be an overestimate by about an order of magnitude. It is somewhat incomprehensible that the authors do explain that when using the RfD for TCM, as recommended by the US EPA, the threshold was not exceeded by measurements taken in Nigeria. They unfortunately did not take this to its logical conclusion: that cancer risks from TCM were in fact calculated to be zero.

The authors present “lifetime incidence rates” (by which they actually mean total lifetime cancer risks) in which lifetime cancer risks are presented per month (Table 4 in the article). It is hard to imagine even a theoretical member of the population only being exposed to DBPs in January, for example, of each year for their entire lifetime, which begs the question: why have these been calculated in this way? Also, it appears that the theoretical population onto which the authors have projected their “total cancer incidence rate” (sic) is permanently divided into those with adult weight and ingestion rates and those with the characteristics of children. This misunderstanding of the lifetime risk assessment framework is exemplified in the sentence, “It was observed that the total lifetime incidence of developing cancer was relatively higher in adults than children.” Do the authors insinuate that one can live a lifetime only as either an adult or a child?

On the basis of their work the authors conclude that DBP formation can be reduced by switching from chlorine to chloramine. This recommendation is highly debatable and absolutely unsupported by the findings presented. While such a switch might reduce the presence of regulated THMs, it is entirely plausible—depending on the characteristics of the raw water and the methods employed—that an increase in unregulated DBPs might also result. The more than six hundred DBPs that have been identified [22, 23] represent only a small fraction of the total organic halides present in chlorinated drinking water [24] and relatively few of those chemicals have been adequately characterised in terms of occurrence. Fewer still have been assessed in terms of their potential effects on human health [23]. Focusing on specific DBPs in the absence of a mechanistic explanation or a true putative agent in the DBP mixture may result in inappropriate or expensive decisions being made in favour of alternative disinfection treatments that may present other health risks, a point convincingly made elsewhere [25]. It is notable, too, that cytotoxicological and genotoxicological studies of various unregulated DBPs have shown that many may be considerably more toxic to humans than THMs [23, 26–28].

In addition to the abovementioned conceptual and methodological limitations in study design and erroneous use of available dose-response data, the conclusions of this study are a particular cause of concern. They may cause unwarranted alarm among the public and potentially lead to poor decisions being made in sourcing, treatment, and provision of drinking water in an environment where good decisions are critical in furthering public health. The authors describe the microbial contamination of drinking water in sub-Saharan Africa as commonplace and presenting “a significant threat to public health.” In the light of this very acute threat to health from microbial contamination, it ought to surprise the readership of this journal greatly that the authors did not take more care to balance this with what could be relatively marginal health risks from DBPs. While it is true that they do not advocate a reduction in disinfection, the authors do suggest that changes to practices at the DWTP may have been responsible for reduced THM concentration in their second sampling period. The real question of interest is what impact these changes to chlorination practices had on chlorine residuals throughout the distribution network and on the efficacy of the disinfectants in terms of preventing very serious risks of spreading microbial disease in the populations supplied.
